# An App-Based Intervention for Caregivers to Prevent Unintentional Injury Among Preschoolers: Cluster Randomized Controlled Trial

**DOI:** 10.2196/13519

**Published:** 2019-08-09

**Authors:** Peishan Ning, Peixia Cheng, David C Schwebel, Yang Yang, Renhe Yu, Jing Deng, Shukun Li, Guoqing Hu

**Affiliations:** 1 Department of Epidemiology and Health Statistics Xiangya School of Public Health Central South University Changsha China; 2 Department of Psychology University of Alabama at Birmingham Birmingham, AL United States; 3 Department of Biostatistics College of Public Health and Health Professions, Emerging Pathogen Institute University of Florida Gainesville, FL United States; 4 Information and Network Center Central South University Changsha China

**Keywords:** unintentional injury, preschoolers, cluster randomized controlled trial, app, mobile health, intervention

## Abstract

**Background:**

App-based interventions have the potential to reduce child injury in countries with limited prevention resources, but their effectiveness has not been rigorously examined.

**Objective:**

This study aimed to assess the effectiveness of an app-based intervention for caregivers of preschoolers to prevent unintentional injury among Chinese preschoolers.

**Methods:**

A 6-month cluster randomized controlled trial was conducted from December 2017 to June 2018. Recruitment was conducted through preschools, which were randomly allocated to either the control group (ie, app-based parenting education excluding unintentional injury prevention) or the intervention group (ie, app-based parenting education including unintentional injury prevention). A total of 2920 caregivers of preschoolers aged 3-6 years from 20 preschools in Changsha, China, were recruited offline through the schools. The primary outcome was unintentional injury incidences among preschoolers in the past 3 months; this measure was assessed through an online caregiver-report at the baseline visit and at 3-month and 6-month follow-up visits. Secondary outcome measures included caregivers’ self-reported attitudes and behaviors concerning child supervision during the last week. Generalized estimating equations (GEEs) were used to assess the effectiveness of the app-based intervention on responses at 3 and 6 months after adjusting for sociodemographic variables, baseline level of the outcome variable, and engagement with interventions in the assigned group. All analyses were intention-to-treat. A per-protocol sensitivity analysis was also conducted.

**Results:**

In total, 1980 of the 2920 caregivers completed the study. The mean age of participants was 32.0 years (SD 5.5) and 68.99% (1366/1980) of them were female. During the 6-month follow-up visit, unintentional injury incidence did not change significantly in either group: incidence in the intervention group went from 8.76% (94/1073) to 8.11% (87/1073), *P*=.59; incidence in the control group went from 9.4% (85/907) to 7.5% (69/907), *P*=.15. The changes did not differ between the groups (odds ratio [OR] 1.14, 95% CI 0.80-1.62). Changes in the average score in attitude concerning unintentional injury prevention were also similar between the groups (B .05, 95% CI -0.03 to 0.13). Changes in unintentional injury prevention behaviors were greater in the intervention group than in the control group after the intervention (B .87, 95% CI 0.33-1.42). Analyses of individual injury prevention behaviors showed that the intervention reduced three risky behaviors: unsafe feeding of children (OR 0.73, 95% CI 0.60-0.89); incorrectly placing children in cars (OR 0.73, 95% CI 0.57-0.93); and allowing children to ride bicycles, electric bicycles, or motorcycles unsupervised (OR 0.80, 95% CI 0.64-0.99). The intervention also improved scores on three safety-focused behaviors: testing water temperature before giving children a bath (OR 1.26, 95% CI 1.05-1.52); properly storing sharp objects (OR 1.24, 95% CI 1.01-1.52); and safely storing medicines, detergents, and pesticides (OR 1.24, 95% CI 1.02-1.51).

**Conclusions:**

The app-based intervention did not reduce unintentional injury incidence among preschoolers but significantly improved caregivers’ safety behaviors. This app-based intervention approach to improve caregiver behaviors surrounding child injury risk offers promise to be modified and ultimately disseminated broadly.

**Trial Registration:**

Chinese Clinical Trial Registry ChiCTR-IOR-17010438; http://www.chictr.org.cn/showproj.aspx?proj=17376 (Archived by WebCite at http://www.webcitation.org/75jt17X84)

**International Registered Report Identifier (IRRID):**

RR2-10.1186/s12889-018-5790-1

## Introduction

Unintentional injuries are a major public health threat to children worldwide. In 2017, it is estimated that over 191,000 children under 5 years of age died from unintentional injuries, with 79% of deaths occurring in low- and middle-income countries (LMICs) [1]. Moreover, nonfatal childhood injuries lead to substantial economic burden and long-term adverse consequences, including physical disability, cognitive or social impairment, and lower educational achievement [2,3].

Previous studies [4-6] indicate that lack of safe parenting behavior, inadequate caregiver perception of risks for child injury, and low adoption of safety equipment usage contribute to the occurrence of child unintentional injury. Parenting interventions to promote child safety have proven effective in high-income countries (HICs) [7] but are not commonly implemented in LMICs like China [8,9].

Mobile health (mHealth)-based interventions offer an opportunity to deliver parenting interventions broadly and cost-effectively, as the usage of mobile phones with expanded and advanced functions (ie, smartphones) rapidly becomes commonplace worldwide. A recent review by Omaki et al [10] provides evidence for the effectiveness of technology-based interventions to improve unintentional injury-prevention behaviors. To date, however, only a few mobile phone app interventions are available to assist parents in preventing unintentional child injuries, and most are not based in theory or are insufficiently tested in rigorous randomized trials [11]. Tests of these interventions were conducted in HICs [12-15] and generally involved assessments of knowledge, perception, and behavioral outcomes with relatively small sample sizes [12,14] (eg, the sample sizes in studies by Gielen et al [12] and Burgess et al [14] were 498 and 742, respectively). None have used actual injury events as the primary outcome indicator [12-15].

This cluster randomized controlled trial (RCT) study aims to evaluate the 6-month effectiveness of a theory-driven, app-based mobile phone intervention for caregivers in preventing unintentional injury among Chinese preschoolers. The study also evaluated whether the intervention improved caregivers’ attitudes concerning injury and their behaviors to promote child safety.

## Methods

### Study Design

A single-blinded, cluster RCT with 1:1 allocation ratio and a follow-up period of 6 months was conducted from December 2017 to June 2018 in Changsha, China. We chose cluster randomization to avoid contamination within the same preschools. The protocol (see Multimedia Appendix 1) was approved by the Ethics Committee of Xiangya School of Public Health, Central South University, Changsha, China (approval number: XYGW-2017-02) and has been published elsewhere [16]. All participants provided informed consent online. This report follows the Consolidated Standards of Reporting Trials (CONSORT) 2010 statement: extension to cluster randomized trials (see Multimedia Appendix 2) [17].

### Participant Recruitment

The recruitment of study participants was based on preschool children aged 3-6 years. We limited the study to preschools with at least 100 students to increase recruitment efficiency. Eligible preschools were randomly selected and contacted by researchers with an official invitation letter along with information about the project. In total, 28 eligible preschools were contacted and 20 agreed to participate (71% participation rate).

All primary caregivers who owned mobile phones and had a preschooler who was 3-6 years old and who was enrolled in a participating preschool were eligible for the study. Primary caregivers were defined as parents, grandparents, other family members, friends of the family, or babysitters and nannies who served as primary caregivers of preschoolers [18]. Teachers from the schools were excluded. One teacher was recruited at each participating preschool to inform eligible caregivers about the study via existing school-family communication channels, including social media platforms (ie, WeChat and QQ), school apps, printed handouts, and oral notification.

Caregivers who agreed to participate received introductory materials about the project. Upon downloading the app, which was developed by the research team and named Bao Hu San (ie, protective umbrella), caregivers viewed and completed online informed consent. Consenting participants then completed an online baseline survey addressing demographic characteristics of caregivers and their children, attitudes toward child injury prevention, supervision behaviors in the last week, and information about any unintentional injury that had occurred to their child during the prior 3 months.

### Sample Size

To obtain adequate power, we calculated the sample size based on the following criteria: baseline unintentional injury incidence of 23% among preschoolers in the past 3 months, effect size (ie, incidence rate ratio) of 0.75 between intervention and control groups, cluster size of 140 children per preschool, and intraclass correlation (ICC) of .005 [19]. Under these criteria, a total of 2626 participants in 20 schools—10 schools per arm—would achieve power of 80% at a .05 significance level, assuming 10% loss to follow-up.

### Randomization Scheme

To avoid potential confounding from the type of preschool, we stratified randomization by type of preschools to reach five public and five private schools per arm. Randomization was performed by an independent (ie, masked) researcher using SAS 9.2 software (SAS Institute).

### Control Group

The control group completed Bao Hu San, an app-based parenting education program developed by the research team. The app trained parents concerning pediatric disease risks and parenting skills but excluded explicit information about unintentional child injury prevention.

### Intervention Group

The intervention group received all content that the control group received using the same app, Bao Hu San, but were also exposed to additional researcher-developed components that focused specifically on unintentional child injury prevention.

### App Design

**Figure 1:**
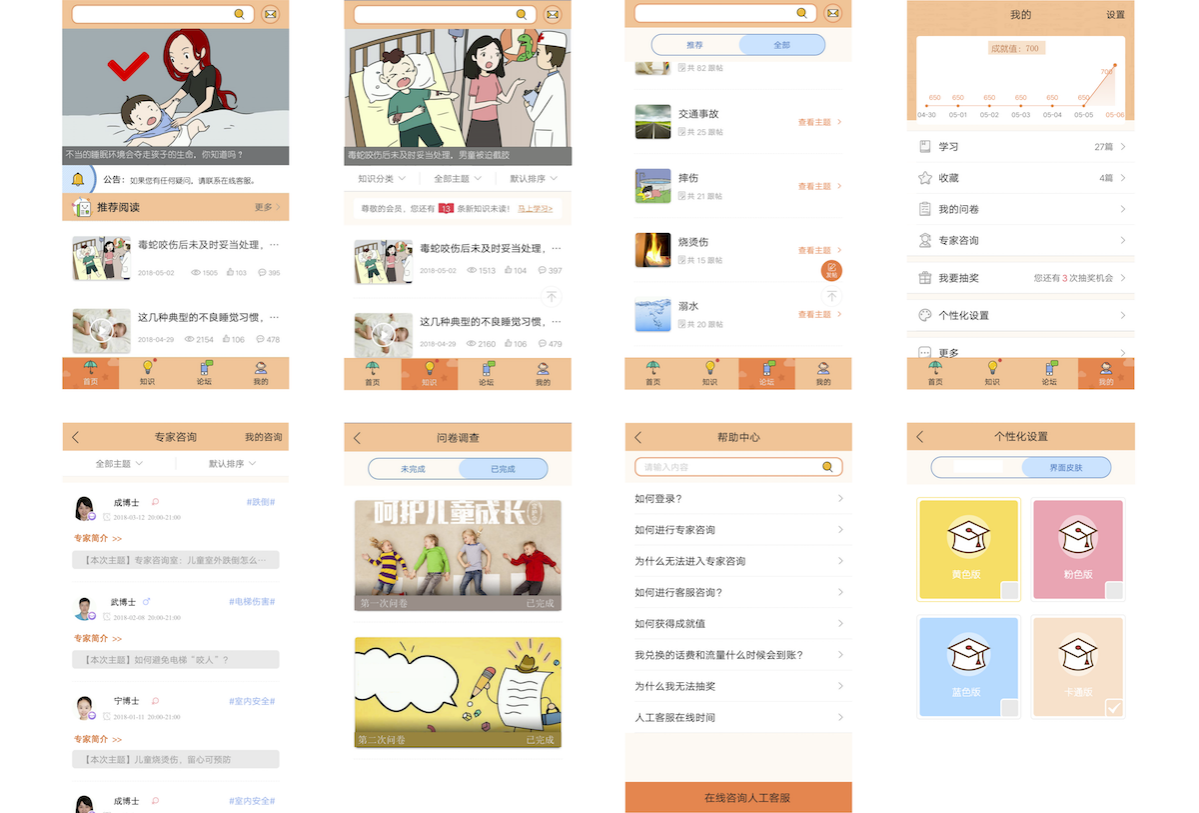
Home page of the app intervention. The app home page as translated into English appears in Figure MA3-1 in Multimedia Appendix 3. Eight images within the figure were derived from the app Bao Hu San, which was developed by the research team for unintentional injury prevention among preschool students and was tested in this trial.

The app components consisted of four active modules: (1) content learning, including lessons to teach caregivers basic knowledge about parenting skills through short written statements with pictures, cartoon vignettes, video testimonials, and interactive games; (2) interaction, containing three submodules to support communication among users (ie, study participants) and between users and professionals; (3) survey and feedback, namely the questionnaire module to collect data online; and (4) personal modules, allowing participants to select the color of the interface in their app according to their preferences (eg, pink, yellow, and blue) (see Figure 1).

The four active modules were available for both the intervention and control groups and differed in two ways. First, the content learning module provided unintentional injury prevention knowledge for the intervention group, in addition to providing pediatric disease risk and parenting skills content available to both groups. Second, communication in the interaction module with injury prevention professionals was only available to the intervention group.

These injury prevention modules were developed based on the principles of the Theory of Planned Behavior (TPB), the Haddon Matrix, and the Framework for the Rational Analysis of Mobile Education (FRAME) model [20-22]. The TPB interventions were designed to target one or more determinants of behavior: attitude, subjective norms, or perceptions of behavioral control. According to health behavior change theory [20], changes in attitudes, subjective norms, and/or perceptions of behavioral control should lead to changes in behavioral intentions, offer adequate perceived control over the behavior, and ultimately lead to behavior change.

The Haddon Matrix is a classical theoretical framework to describe the occurrence of an injury event from three phases (ie, pre-event, event, and post-event) and four factors (ie, host, agent or vehicle, physical environment, and social environment) [21].

As a comprehensive model to develop mobile learning apps, the FRAME model allows researchers to consider all relevant components of users’ learning in the development of the app [22]. Among the components that receive focus are usability of the device and app, capacities of the learners, and social interaction between users. We followed the FRAME model when developing the app and its multiple components; we implemented the FRAME model through results from a needs assessment that consisted of a series of focus groups plus online surveys among key stakeholders, including preschool teachers and caregivers of preschoolers [23].

We implemented several strategies to encourage participants in both the intervention and control groups to actively use the app: (1) awarding of app-based virtual currency rewards, (2) awarding of additional rewards by lottery for participants who continuously logged in to the app for 7 consecutive days, (3) offering monthly rewards to the caregivers of the preschool classes with the most frequent log-ins, and (4) sending of regular reminders about app engagement from preschool teachers. Detailed descriptions about the intervention are available elsewhere [16].

Routine parenting educational activities offered by the preschool or other institutions (eg, children’s hospitals and communities), whether related to unintentional injury prevention or not, were permitted in both groups. All preschools involved in the study received honoraria at months 1, 3, and 6.

### Outcome Measurements

#### Primary Outcome

The primary outcome was unintentional injury incidence among preschoolers in the prior 3 months, as collected at the baseline visit and at 3-month and 6-month follow-up visits through online surveys. Unintentional injuries were defined according to the International Classification of Diseases, 10th Revision [24]. Similar to previous studies [25], injury events were defined as incidents meeting any of the following criteria: (1) child receives medical treatment by a doctor or other medical professional following an injury; (2) child receives first aid by a family member, teacher, or other nonmedical staff following an injury (eg, medication, massage, or hot compress); and/or (3) child is restricted from school or other activities, or is kept in bed or rest for more than a half day following an injury.

Unintentional injury incidence in the prior 3 months was calculated as follows:

(Number of preschoolers experiencing an unintentional injury in the prior 3 months) / (Total number of children supervised by recruited caregivers) × 100%

#### Secondary Outcomes

Secondary outcomes, as outlined a priori in the study protocol [16], included the following: (1) caregivers’ attitudes toward unintentional injury prevention and safety behaviors among preschoolers; (2) economic losses due to unintentional injury, including direct economic costs (eg, medical treatment expenses) and indirect economic costs (eg, caregiver’s economic loss from being off work); and (3) the incremental cost-effectiveness ratio (ICER) for the app-based unintentional injury intervention, calculated as the cost difference between the intervention group and the control group divided by the difference in the number of unintentional injury events among children in the two arms. We did not analyze economic losses because some respondents could not remember the amount of medical expenses and indirect costs due to child unintentional injury, and others viewed medical expenses as sensitive private information they were not willing to report. We also excluded the ICER from our final analysis because changes in unintentional injury incidence were not statistically significant.

Both caregiver attitudes toward child injury prevention and frequency of engaging in injury prevention behaviors served as secondary outcomes. Each was measured via self-report using a 4-point scale: attitudes were reported as *completely agree, partly agree, not sure*, or *not at all agree*; behavior was reported as *0 times in the past week, 1-2 times in the past week, 3-5 times in the past week*, or *≥* *6 times in the past week*. The total attitude score ranged from 2 to 8 points. The four categories were then quantified as 4, 3, 2, and 1 for attitudes and risky behaviors and as 1, 2, 3, and 4 for safe behaviors, so that higher scores in both cases reflected greater awareness of, or health-promoting behavior toward, injury prevention. The total behavior score ranged from 15 to 60 points.

**Items assessing self-reported caregiver attitudes concerning child injury and health behaviors to prevent unintentional child injury.**

Two items assessing attitudes:

1. Preventability of preschooler unintentional injury
2. Self-efficacy to keep child safe from unintentional injuries

15 items (nine for risky behaviors and six for safe behaviors):

Risky behaviors:

1. Leaving child alone in the home
2. Leaving child alone in the bathroom while bathing
3. Criticizing child when they are eating or drinking, creating a choking or suffocation risk
4. Giving child whole or large pieces of food that create choking risk
5. Placing child in the front seat while riding in a car
6. Not using child restraints while riding in a car
7. Letting child ride a bicycle, electric bicycle, or motorcycle unsupervised
8. Letting child take an escalator alone
9. Letting child contact unfamiliar or aggressive animals

Safe behaviors:

1. Holding child’s hand while crossing the street
2. Testing water temperature before giving child a bath
3. Placing hot substances and lighters where children cannot reach
4. Placing sharp objects where children cannot reach
5. Storing medicines, detergents, and pesticides where children cannot reach
6. Wearing safety equipment when child rides a bicycle, electric bicycle, or motorcycle

The caregiver’s overall attitudes and behaviors concerning unintentional injury prevention were calculated as the sum of two and 15 items, respectively (see Textbox 1). All items were adapted from previous studies and have demonstrated psychometric reliability and validity [26,27]. Prior to use in our study, we conducted a brief validation study of the instrument after translating it into Chinese. Among a sample of 100 caregivers not enrolled in the larger study, test-retest reliability over a 1-week interval for the sum score of the two items assessing attitudes and the 15 items assessing behaviors was r_s_=0.69 and r_s_=0.64, respectively.

#### Post Hoc Outcomes

We define the incidence of each risky or safe behavior listed in Textbox 1 as follows:

(Number of caregivers reporting a specific risky or safe behavior in the past week) / (Total number of participating caregivers) × 100%

This allows us to focus on the presence or absence of each of the risky and safe behaviors. In total, 15 item scores were calculated, corresponding to the 15 risky and safe behaviors listed in Textbox 1. We also considered engagement-level data. These data were recorded automatically through electronic strategies embedded in the app and included the number of log-ins, the length of time using the app at each log-in, the number of knowledge segments (eg, short written statements with pictures, cartoon vignettes, video testimonials, and interactive games) studied and bookmarked, and the number of posted comments.

### Statistical Analysis

The chi-square test and the Wilcoxon rank-sum test were used to examine differences in demographic characteristics between the two trial groups. The generalized estimating equation (GEE) was used to assess the effectiveness of the app-based intervention on responses at 3 and 6 months after adjusting for sociodemographic variables, being taught about injury prevention in the past 3 months, frequency of using parenting apps, baseline level of the outcome variable, and the level of engagement with the app in the assigned group. A logistic link function was used for dichotomous outcomes and identity link was used for continuous outcomes (see the Statistical Models Section in Multimedia Appendix 3). All analyses were intention-to-treat. Missing values were imputed using the expectation-maximization algorithm before the GEE analysis. To test the robustness of the results, a per-protocol sensitivity analysis was conducted.

For the evaluation of parenting-related attitudes concerning, and behaviors to prevent unintentional child injury, we first examined mean differences in total scores between the two groups. Incidence differences in individual behaviors were examined if the total scores differed significantly between groups.

Group assignment was masked during data analysis. All statistical analyses were performed using SAS 9.2 (SAS Institute). All statistical tests were 2-sided tests at the .05 significance level.

## Results

### Baseline Characteristics of Participants

**Figure 2:**
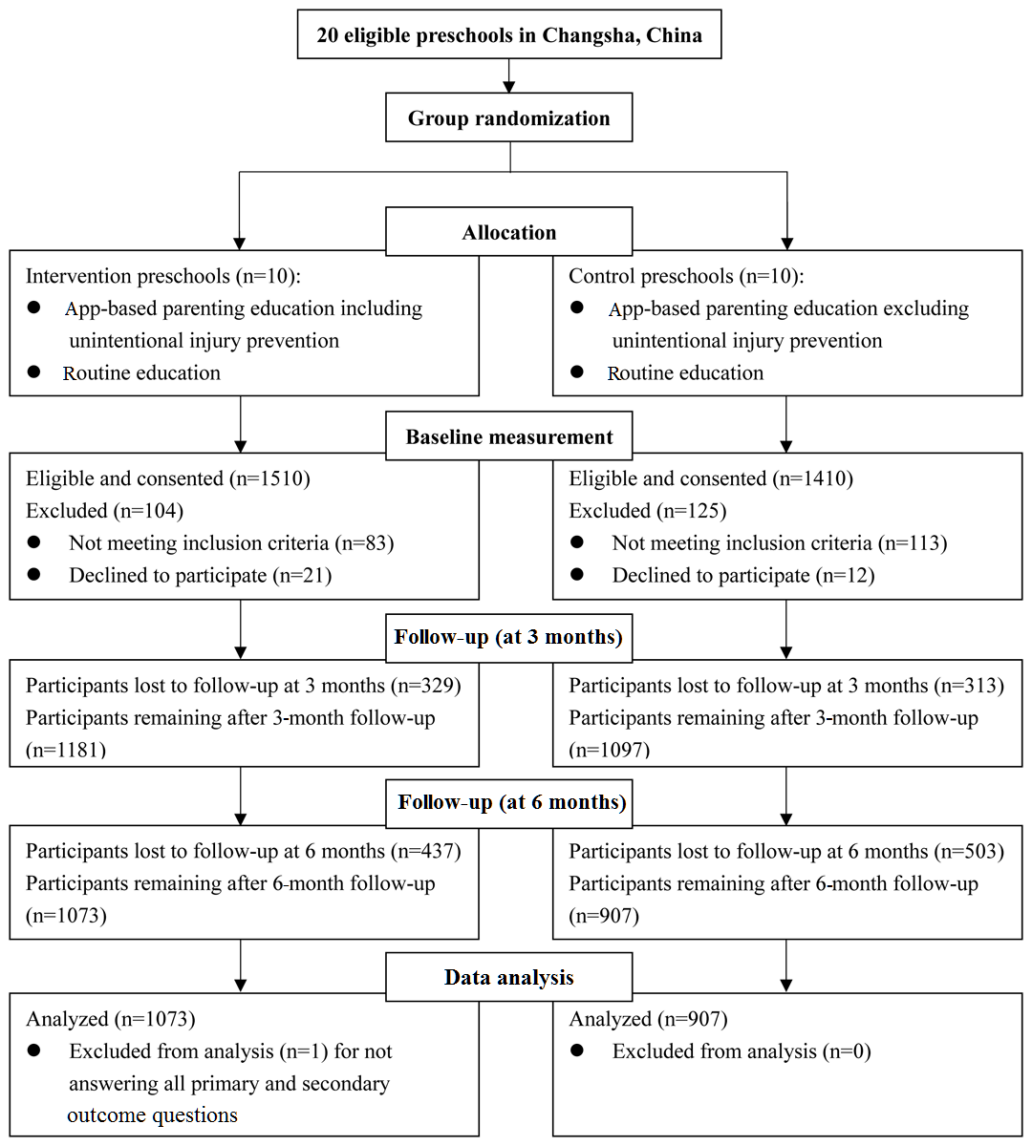
Flow diagram of the study.

In total, 1980 of the consented 2920 caregivers (67.81%) completed the surveys at baseline and at both 3- and 6-month follow-up visits. Of the 1980 who completed all surveys, 1073 (54.19%) were in the intervention group and 907 (45.81%) were in the control group (see Figure 2). Caregivers in the intervention group were significantly younger (32.9 vs 33.6 years, *P*=.01), more often male than female (371/1073, 34.58% vs 243/907, 26.8%, *P*<.001), had different household income levels (*P*=.004), and were more likely to have been taught about injury prevention in the past 3 months (677/1073, 63.09% vs 491/907, 54.1%, *P*<.001) compared to caregivers in the control group (see Table 1). Other between-group demographic differences were not significant. The imbalanced baseline characteristics were adjusted for in all subsequent analyses of intervention effectiveness.

**Table 1:** Baseline characteristics of study participants who completed baseline surveys and both 3-month and 6-month follow-up surveys.

Characteristics		Total (N=1980)	Intervention group (N=1073)	Control group (N=907)	*P* value
Adult age in years, mean (SD)		32.0 (5.5)	32.9 (5.0)	33.6 (6.0)	.01
**Adult gender, n (%)**					
	Male	614 (31.0)	371 (34.6)	243 (26.8)	<.001
	Female	1366 (69.0)	702 (65.4)	664 (73.2)	
Child age in years, mean (SD)		4.5 (0.9)	4.5 (1.0)	4.5 (0.9)	.68
**Child gender, n (%)**					
	Male	1013 (51.2)	536 (50.0)	477 (52.6)	.24
	Female	967 (48.8)	537 (50.0)	430 (47.4)	
**Adult education, n (%)**					
	Junior high school or below	118 (6.0)	64 (6.0)	54 (6.0)	.93
	High school	475 (24.0)	261 (24.3)	214 (23.6)	
	College and above	1387 (70.1)	748 (69.7)	639 (70.5)	
**Household income per capita per month in Yuan, n (%)**					
	<1500	51 (2.6)	37 (3.4)	14 (1.5)	.004
	1500-3499	366 (18.5)	213 (19.9)	153 (16.9)	
	3500-5499	1339 (67.6)	634 (59.1)	705 (77.7)	
	≥5500	224 (11.3)	189 (17.6)	35 (3.9)	
**Frequency of using parenting apps, n (%)**					
	More than once a day	170 (8.6)	96 (8.9)	74 (8.2)	.53
	Every two or three days	224 (11.3)	127 (11.8)	97 (10.7)	
	Once a week	253 (12.8)	143 (13.3)	110 (12.1)	
	Every two weeks or less	1333 (67.3)	707 (65.9)	626 (69.0)	
**Taught about injury prevention in the past 3 months, n (%)**					
	Yes	1168 (59.0)	667 (63.1)	491 (54.1)	<.001
	No	812 (41.0)	396 (36.9)	416 (45.9)	

Participants who did not complete the study (n=940) were similar to those who completed the study on several baseline characteristics: mean child age, *P*=.54; child gender, *P*=.83; household income, *P*=.50; and frequency of using parenting apps, *P*=.65. However, the caregivers who withdrew were significantly younger, more often female, had lower education levels, and were previously taught about injury prevention less often than the completers (see Table MA3-1 in Multimedia Appendix 3).

### Intervention Engagement

Over the 6-month study period, the mean number of log-ins for all participating caregivers was 37.7 (SD 66.7) times, with an average of 39.3 (SD 42.5) knowledge segments learned, 1.8 (SD 7.3) knowledge segments bookmarked, and 29.9 (SD 67.1) comments posted. Overall log-in time duration using the app in the study period averaged 150.5 minutes (SD 239.7) (see Table MA3-2 in Multimedia Appendix 3). The intervention group on average had a higher number of knowledge segments studied (45.0 vs 32.6, *P*=.001) and a longer overall duration of app usage (161.2 vs 137.8 minutes, *P*=.03) than the control group, but no differences were found between the groups for other engagement indicators.

### Unintentional Injury Incidence

After the 6-month intervention, unintentional injury incidence in the past 3 months decreased from 8.76% (94/1073) at baseline to 8.11% (87/1073) in the intervention group (*P*=.59), and decreased from 9.4% (85/907) to 7.5% (69/907) in the control group (*P*=.15) (see Table 2). The changes were not statistically significant between groups after adjusting for covariates: sociodemographic variables, outcome measures at baseline, and engagement to the intervention (odds ratio [OR] 1.14, 95% CI 0.80-1.62).

**Table 2:** Results for primary and secondary outcomes based on generalized estimating equations (GEEs).

Outcome measure		Intervention group (N=1073)	Control group (N=907)	Adjusted OR^a^ or B^b^ (95% CI)^c^	*P* value
**Unintentional injury incidence, % (95% CI)**						
	Baseline	8.8 (7.0-10.6)	9.4 (7.4-11.4)	—^d^	—
	3-month	7.7 (6.1-9.3)	7.1 (5.3-8.9)	1.16 (0.82 to 1.63)^a^	.41
	6-month	8.1 (6.5-9.7)	7.5 (5.7-9.3)	1.14 (0.80 to 1.62)^a^	.47
**Score of attitudes toward unintentional child injury prevention, mean (95% CI)**					
	Baseline	6.6 (6.6-6.7)	6.6 (6.5-6.6)	—	—
	3-month	6.6 (6.6-6.7)	6.6 (6.6-6.7)	0.03 (-0.06 to 0.13)^b^	.48
	6-month	6.8 (6.7-6.8)	6.7 (6.6-6.7)	-0.05 (-0.14 to 0.04)^b^	.31
**Score of behavior to prevent unintentional child injury, mean (95% CI)**					
	Baseline	47.0 (46.7-47.4)	47.2 (46.8-47.6)	—	—
	3-month	48.7 (48.4-49.1)	48.4 (48.0-48.8)	0.40 (-0.14 to 0.94)^b^	.15
	6-month	48.9 (48.5-49.3)	48.0 (47.6-48.5)	0.87 (0.33 to 1.42)^b^	.002

^a^OR: odds ratio.

^b^B: regression coefficient.

^c^Odds ratio and regression coefficient are presented for the intervention effect from the generalized estimating equation (GEE) after adjusting for sociodemographic variables (ie, caregiver’s age, gender, education level, household income, frequency of using parenting apps, and recent learning about child injury prevention; and child’s age and gender), outcome variables at baseline, and engagement with the interventions in the assigned group (ie, number of log-ins, length of time using the app at each log-in, number of knowledge segments studied, number of knowledge segments bookmarked, and number of posted comments).

^d^Reference group.

### Attitudes Concerning Child Unintentional Injury and Behaviors to Prevent Injuries

The mean caregiver total attitude score changed from 6.6 (95% CI 6.6-6.7) at baseline to 6.8 (95% CI 6.7-6.8) at the 6-month follow-up for the intervention group, and from 6.6 (95% CI 6.5-6.6) to 6.7 (95% CI 6.6-6.7) for the control group (see Table 2). Mean changes in the total score were not significant between the two groups after adjusting for the covariates (B .05, 95% CI -0.03 to 0.13).

The mean changes in total caregiver behavior scores to prevent unintentional child injury did differ between the control and intervention groups (*P*=.002) after the 6-month intervention. After adjusting for sociodemographics, baseline outcomes, and engagement factors, behavior scores increased from 47.0 (95% CI 46.7-47.4) to 48.9 (95% CI 48.5-49.3) for the intervention group and from 47.2 (95% CI 46.8-47.6) to 48.0 (95% CI 47.6-48.5) for the control group (B .87, 95% CI 0.33-1.42) (see Table 2).

**Figure 3:**
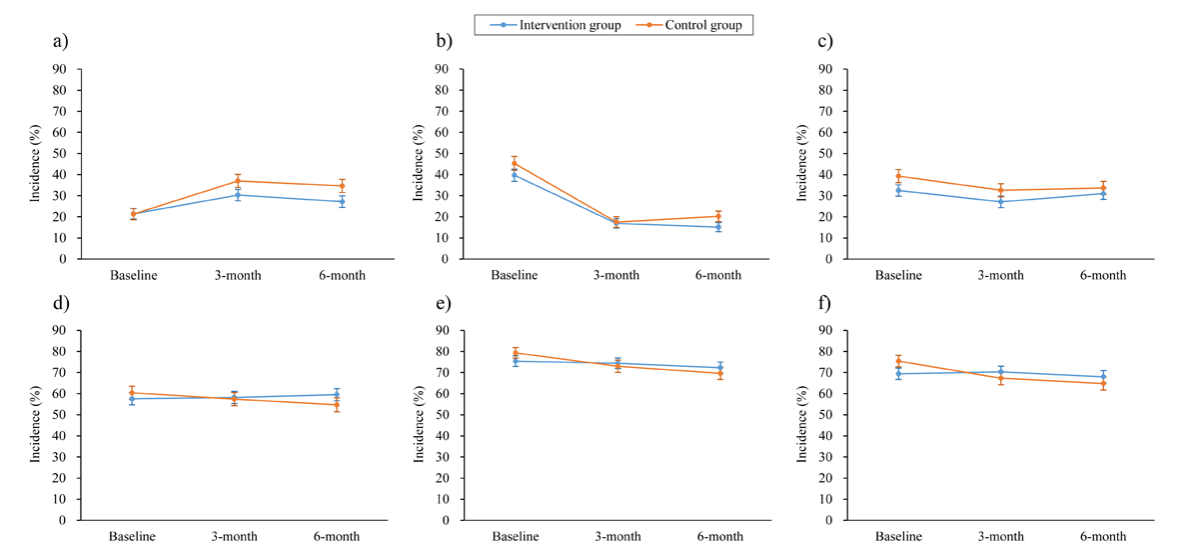
Incidence of caregiver parenting behaviors to prevent unintentional child injury in the past week. a) Giving child a whole or large piece of food that creates a choking risk; b) Placing child in the front seat while riding in a car; c) Letting child ride a bicycle, electric bicycle, or motorcycle unsupervised; d) Testing water temperature before giving child a bath; e) Placing sharp objects where children cannot reach; f) Storing medicines, detergents, and pesticides where children cannot reach. Only those parenting behaviors with significant changes between intervention and control groups are included here.

When we examined individual injury prevention behavior scores, we detected significant differences between the groups in the changes of the scores from baseline to 6-month follow-up for two risky behaviors: giving children whole or large pieces of food (OR 0.73, 95% CI 0.60-0.89) and placing children in the front seat while riding a car (OR 0.73, 95% CI 0.57-0.93). We also detected significant between-group differences in score changes for three safe behaviors: testing the water temperature before giving children a bath (OR 1.26, 95% CI 1.05-1.52); placing sharp objects where children cannot reach them (OR 1.24, 95% CI 1.01-1.52); and storing medicines, detergents, and pesticides where children cannot reach them (OR 1.24, 95% CI 1.02-1.51) (see Figure 3). Another risky behavior was reduced at the 3-month follow-up visit: letting child ride a bicycle, electric bicycle, or motorcycle unsupervised (OR 0.80, 95% CI 0.65-0.99), but this behavior did not demonstrate significant change at the 6-month follow-up visit (see Table MA3-3 in Multimedia Appendix 3).

Results from the sensitivity analysis using per-protocol analysis were similar to the presented intention-to-treat analysis (see Table MA3-4 in Multimedia Appendix 3).

## Discussion

### Principal Findings

To our knowledge, this study represents the first cluster RCT testing the effectiveness of a theory-driven app-based intervention to prevent unintentional child injury through improved parenting of preschoolers in China. We found that following 6 months of using the app, caregivers in the intervention group did not report significantly different rates of unintentional injury incidence among their preschoolers compared to caregivers in the control group, nor did caregivers’ attitudes toward unintentional child injury prevention differ across the groups. We did discover differences in caregivers’ self-reported injury prevention behaviors, with caregivers in the intervention group reporting more increases in injury prevention behaviors after using the app for 6 months compared to those in the control group.

### Interpretation

Despite findings that suggest parent behaviors changed after engaging in the intervention, results did not support our hypothesis that the app would reduce unintentional child injury incidence. The results we found concerning changes in parenting behavior agree with previous publications in HICs [12-15]. The nonsignificant results for unintentional child injury incidence may be a result of a few factors. First, isolated parenting behaviors do not prevent all child injuries. When children are supervised by others, such as teachers, parent behavior changes will likely have minimal influence. Further, the social and physical environments the child engages within are likely to be influenced minimally by caregivers but are likely to influence child injury risk to some degree [20,28]. A second factor that may have influenced our nonsignificant results is the fact that child injuries leading to serious restriction in activity or professional medical care are comparatively low. As is common in the field [1], injuries were relatively infrequent among our sample: incidence over the past 3 months was 9.14% (ie, at baseline, 181 out of 1980 participants experienced an injury in the past 3 months). Although we recruited a large sample size and examined injuries over a 6-month period, we may not have accumulated sufficient events to statistically detect the intervention effect for a minor effect size (OR 1.16 in this study).

Third, the effectiveness of any app-based interventions depends on the users’ engagement [29]. Participants in the intervention group spent an average of 2.7 hours using the app, during which they studied an average of 45 knowledge segments and posted an average of 31.5 comments (see Table MA3-2 in Multimedia Appendix 3). There is no standard to dictate what extent of app engagement might yield knowledge transmission or behavior change. However, results of participant engagement from our study are consistent with another study reporting null results [30], and engagement was significantly lower than in studies reporting significant effects (eg, 13.1 hours of engagement in an effective collaborative care intervention for violence risk behaviors, substance use, and posttraumatic stress and depressive symptoms among injured adolescents [31]).

One other finding is noteworthy: to our surprise, we did not detect significant changes in parenting attitude scores between the groups after the 6-month intervention. This finding may reflect a ceiling effect: caregivers in both groups already had high attitude scores at baseline and, therefore, a significant change was difficult to achieve. For example, the proportion of caregivers reporting low self-efficacy to keep children safe from unintentional injuries was only 3.73% (40/1073) in the intervention group and 3.8% (34/907) in the control group at baseline (see Figure MA3-2 in Multimedia Appendix 3).

### Implications

Our findings have two implications. First, in combination with previous findings [12-15], our results suggest that a mobile phone-based app that is grounded in behavior change theory might ultimately be effective to improve child injury prevention behaviors among caregivers of preschoolers in China. Specifically, in this study we yielded change in self-reported behavior, although we did not yield statistically significant change in injury incidence or caregiver attitudes about safety. These results imply there may be a need for further app development and for empirical research to discover what strategies might be most effective to yield child safety-related behavior change among caregivers and ultimately reduce injury risk among children. For example, refinement of the app might include ways to engage caregivers more intensely and effectively, perhaps through a more elaborate system of points, levels, or rewards. Others have reported increased engagement through attractive multimedia content and features with diversified content and delivery forms (eg, video, audio, games, and progress bars) [32]; increased engagement might lead more effectively to desired behavior change outcomes [29].

Second, app-based interventions might not function well in isolation. Instead, they might be more beneficial if they were integrated into a multi-faceted intervention program that maximizes the benefit of mobile health technology in safety education. For example, our app may be integrated within the Basic Public Health Service Program [33] run by the Chinese government, and then re-evaluated for its value in unintentional child injury prevention at a larger scale.

### Limitations

This study had several limitations. First, our results relied on self-reported data and used a recall time period of the past 3 months for the child injury reports. Such data may be influenced by various biases [34,35]. Second, a few demographic characteristics differed between the participants who withdrew from the study compared to those who completed. Differential withdrawal rates may have led to unexpected biases in our findings. Third, the inclusion criteria of recruiting children only from preschools with more than 100 students may restrict the generalizability of our findings. Such relatively large preschools represent 87.8% (44/361) of all preschools in Changsha, but families that choose smaller preschools for their children may be different in some characteristics relevant to the effectiveness of the intervention.

In addition, we permitted study participants in both groups to attend parenting-related education and learning activities, which might have contaminated our data in some way. However, we felt it unethical to prohibit such educational activities, and randomization suggests that potential biases in our findings due to such contamination would be minimal. Finally, the actual incidence of child unintentional injury in our study was lower than that estimated when we calculated sample size requirements for the study. The withdrawal rate of participants (940/2920, 32.19%) in the study was also higher than expected. Both of these factors led to a sample size that was likely inadequate to detect small incidence or behavior changes. Future studies should consider strategies to retain participants; these might include a more engaging app, more frequent communications and reminders to users, financial incentives for completion of study tasks, and community involvement in the study design and implementation [36-38].

### Conclusions

The app-based intervention for caregivers did not significantly reduce unintentional injury incidence among Chinese preschoolers but did substantially improve caregivers’ behaviors relevant to prevention of unintentional injury. We recommend further refinement and assessment of the app-based intervention to improve its effectiveness in reducing child injury risk in China and other LMICs.

## Appendix

Multimedia Appendix 1 Effectiveness of an app-based intervention for unintentional injury among caregivers of preschoolers: Protocol for a cluster randomized controlled trial.

Multimedia Appendix 2 CONSORT-EHEALTH checklist (V 1.6.1).

Multimedia Appendix 3 Statistical models, appendix figures, and appendix tables.

## Abbreviations

CONSORT: Consolidated Standards of Reporting Trials
FRAME: Framework for the Rational Analysis of Mobile Education
GEE: generalized estimating equation
HIC: high-income country
ICC: intraclass correlation
ICER: incremental cost-effectiveness ratio
LMICs: low- and middle-income countries
mHealth: mobile health
OR: odds ratio
RCT: randomized controlled trial
TPB: Theory of Planned Behavior
