# Supplementary material for: An App-Based Intervention for Caregivers to Prevent Unintentional Injury Among Preschoolers: Cluster Randomized Controlled Trial
Source: JMIR Mhealth Uhealth. 2019 Aug 9;7(8):e13519. doi: 10.2196/13519 (PMC6713040; doi:10.2196/13519)
Supplement: Multimedia Appendix 3 [file mhealth_v7i8e13519_app3.pdf]

1. Statistical Models

A typical logistic regression is of the form

$$\log\frac{P(Y_t=1)}{P(Y_t=0)} = \beta_0 + \beta_1 I_{t=6} + \beta_2 I_{t=6}G + \beta_3(1 - I_{t=6})G + \beta_4 Y_0 + \boldsymbol{\beta}_5{}'\boldsymbol{X},$$

where Y_t indicates presence (1) or absence (0) of unintentional child injury in the past 3 months at month t, with $t = 0, 3, 6$; $I_{t=6}$ indicates month 6 ($I_{t=6} = 1$) or month 3 ($I_{t=6} = 0$) and *G* indicates the assigned group (intervention=1, control=0). Y_3 and Y_6 are the outcomes to be modeled, and Y_0 is adjusted for as a covariate. Other covariates are coded in vector $\boldsymbol{X}$. Odds ratios (ORs) e^{β_2} and e^{β_3} represent the intervention effects at month 3 and 6, respectively. As the trial was randomized and the adjustment for Y_0 captures potential imbalance of risk of unintentional injury at baseline, e^{β_2} and e^{β_3} also represent the intervention effects on changes in the risk from baseline to month t.

For continuous outcomes, we fit an ordinary linear regression of a similar form

$$Y_t = \beta_0 + \beta_1 I_{t=6} + \beta_2 I_{t=6}G + \beta_3(1 - I_{t=6})G + \beta_4 Y_0 + \boldsymbol{\beta}_5{}'\boldsymbol{X}.$$

The intervention effects are represented by the coefficients β_2 and β_3 for months 3 and 6, respectively. This model is equivalent to the regression that explicitly models changes in the outcome between baseline and month t:

$$Y_t - Y_0 = \beta_0 + \beta_1 I_{t=6} + \beta_2 I_{t=6}G + \beta_3(1 - I_{t=6})G + \tilde{\beta}_4 Y_0 + \boldsymbol{\beta}_5{}'\boldsymbol{X},$$

where $\tilde{\beta}_4 = \beta_4 - 1$.

Table MA3-1. Demographic characteristics of withdrawn and completing participants

Characteristics	Withdrawn		Completed		*P*[a]
	n	%	n	%	
**Total**	940	100.0	1980	100.0	
**Adult age, mean (SD), years**		32.2±4.9		33.2±5.5	<.001
**Adult sex**					
Male	240	25.5	614	31.0	.002
Female	700	74.5	1366	69.0	
**Child age, mean (SD), years**		4.5±1.0		4.5±0.9	.54
**Child sex**					
Male	485	51.6	1013	51.2	.83
Female	455	48.4	967	48.8	
**Adult education**					
Junior high school or below	91	9.7	118	6.0	<.001
High school	268	28.5	475	24.0	
University or above	581	61.8	1387	70.1	
**Household income per capita per month, Yuan**					
<1500	34	3.6	51	2.6	.50
1500-3499	177	18.8	366	18.5	
3500-5499	621	66.1	1339	67.6	
≥5500	108	11.5	224	11.3	
**Frequency of using parenting apps**					
More than once a day	76	8.1	170	8.6	.65
Every two or three days	110	11.7	224	11.3	
Once a week	204	21.7	253	12.8	
Every two weeks or less	550	58.5	1333	67.3	
**Taught about injury prevention in the past 3 months**					
Yes	458	48.7	1168	59.0	<.001
No	482	51.3	812	41.0	

[a]: Chi-square test and Wilcoxon rank sum test were used to examine differences in demographic characteristics between withdrawn and completing participants.

Table MA3-2. Engagement in using the unintentional child injury prevention app

Indicator	Total (N=1980)		Intervention group (N=1073)		Control group (N=907)		*P*[a]
	Mean (SD)	Median (QR)	Mean (SD)	Median (QR)	Mean (SD)	Median (QR)	
Number of logins	37.7 (66.7)	15 (36)	38.4 (71.2)	15 (34)	36.8 (61.0)	14 (36)	.58
Number of knowledge segments that were studied	39.3 (42.5)	22 (69)	45.0 (48.6)	23 (82)	32.6 (32.8)	20 (61)	<.001
Number of knowledge segments bookmarked by caregiver	1.8 (7.3)	0 (1)	1.8 (8.3)	0 (1)	1.6 (5.8)	0 (1)	.55
Length of time using the app, minutes	150.5 (239.7)	65.2 (172.2)	161.2 (261.1)	67.1 (182.2)	137.8 (211.2)	62.4 (166.3)	.031
Number of published comments	29.9 (67.1)	2 (32)	31.5 (58.8)	2 (35)	28.0 (75.7)	2 (30)	.25

Abbreviation: SD, Standard deviation; QR: quartile range.

[a]: Z-test examined differences between intervention and control group.

Table MA3-3. Specific behavior to prevent unintentional child injury in the past week

Outcome measure	% (95% CI)		Adjusted OR
	Intervention group	Control group	(95% CI)[a]
**1. Risky factors**			
**Leaving child alone in the home**			
Baseline	8.0 (6.4, 9.6)	8.3 (6.5, 10.1)	
3-month	9.0 (7.2, 10.8)	9.2 (7.2, 11.2)	0.99 (0.72, 1.38)
6-month	11.4 (9.4, 13.4)	10.9 (8.9, 12.9)	1.06 (0.78, 1.43)
**Leaving child alone in the bathroom while bathing**			
Baseline	28.5 (25.8, 31.2)	28.0 (25.1, 30.9)	
3-month	27.2 (24.5, 29.9)	28.6 (25.7, 31.5)	0.95 (0.78, 1.18)
6-month	23.6 (21.1, 26.1)	27.5 (24.6, 30.4)	0.83 (0.67, 1.02)
**Criticizing child when they are eating or drinking, creating choking/suffocation risk**			
Baseline	45.4 (42.5, 48.3)	45.3 (42.0, 48.6)	
3-month	36.5 (33.6, 39.4)	37.6 (34.5, 40.7)	0.97 (0.80, 1.18)
6-month	34.1 (31.4, 36.8)	37.8 (34.7, 40.9)	0.86 (0.71, 1.05)
**Giving child whole or large pieces of food that creates a choking risk**			
Baseline	21.4 (18.9, 23.9)	21.3 (18.6, 24.0)	
3-month	30.3 (27.6, 33.0)	37.0 (33.9, 40.1)	0.76 (0.63, 0.93)*
6-month	27.2 (24.5, 29.9)	34.6 (31.5, 37.7)	0.73 (0.60, 0.89)*
**Placing child in the front seat while riding in a car**			
Baseline	39.7 (36.8, 42.6)	45.3 (42.0, 48.6)	
3-month	16.9 (14.7, 19.1)	17.5 (15.0, 20.0)	1.00 (0.78, 1.27)
6-month	15.1 (12.9, 17.3)	20.2 (17.7, 22.7)	0.73 (0.57, 0.93)*
**Not using child restraints while riding in a car**			
Baseline	43.0 (40.1, 45.9)	38.1 (35.0, 41.2)	
3-month	48.1 (45.2, 51.0)	49.1 (45.8, 52.4)	1.02 (0.84, 1.23)
6-month	43.2 (40.3, 46.1)	46.0 (42.7, 49.3)	0.95 (0.79, 1.14)
**Letting child ride a bicycle, electric bicycle, or motorcycle unsupervised**			
Baseline	32.5 (29.8, 35.2)	39.3 (36.2, 42.4)	
3-month	27.1 (24.4, 29.8)	32.6 (29.5, 35.7)	0.80 (0.65, 0.99)*
6-month	31.0 (28.3, 33.7)	33.7 (30.6, 36.8)	0.93 (0.76, 1.14)
**Letting child take an escalator alone**			
Baseline	23.4 (20.9, 25.9)	27.1 (24.2, 30.0)	
3-month	11.5 (9.5, 13.5)	10.6 (8.6, 12.6)	1.08 (0.80, 1.44)
6-month	10.7 (8.9, 12.5)	12.0 (9.8, 14.2)	0.86 (0.64, 1.16)
**Letting child contact unfamiliar or aggressive animals**			

Baseline	33.1 (30.4, 35.8)	39.8 (36.7, 42.9)	
3-month	4.1 (2.9, 5.3)	2.6 (1.6, 3.6)	1.63 (0.98, 2.73)
6-month	3.9 (2.7, 5.1)	5.7 (4.1, 7.3)	0.69 (0.45, 1.06)
**2. Safe behaviors**			
**Holding child's hand while crossing the street**			
Baseline	84.9 (82.7, 87.1)	87.9 (85.7, 90.1)	
3-month	87.2 (85.2, 89.2)	87.3 (85.1, 89.5)	1.02 (0.78, 1.35)
6-month	84.5 (82.3, 86.7)	83.8 (81.4, 86.2)	1.09 (0.85, 1.41)
**Testing water temperature before giving child a bath**			
Baseline	57.6 (54.7, 60.5)	60.4 (57.3, 63.5)	
3-month	58.2 (55.3, 61.1)	57.4 (54.3, 60.5)	1.07 (0.88, 1.29)
6-month	59.5 (56.6, 62.4)	54.7 (51.4, 58.0)	1.26 (1.05, 1.52)*
**Placing hot substances and lighters where children cannot reach**			
Baseline	70.4 (67.7, 73.1)	75.5 (72.8, 78.2)	
3-month	70.5 (67.8, 73.2)	69.2 (66.3, 72.1)	1.13 (0.92, 1.38)
6-month	67.9 (65.2, 70.6)	66.5 (63.4, 69.6)	1.14 (0.93, 1.38)
**Placing sharp objects where children cannot reach**			
Baseline	75.4 (72.9, 77.9)	79.3 (76.8, 81.8)	
3-month	74.4 (71.9, 76.9)	73.0 (70.1, 75.9)	1.16 (0.94, 1.43)
6-month	72.3 (69.6, 75.0)	69.6 (66.7, 72.5)	1.24 (1.01, 1.52)*
**Storing medicines, detergents, and pesticides where children cannot reach**			
Baseline	69.4 (66.7, 72.1)	75.4 (72.7, 78.1)	
3-month	70.3 (67.6, 73.0)	67.3 (64.2, 70.4)	1.24 (1.01, 1.52)*
6-month	68.0 (65.3, 70.7)	64.8 (61.7, 67.9)	1.24 (1.02, 1.51)*
**Wearing safety equipment when child rides a bicycle, electric bicycle, or motorcycle**			
Baseline	44.9 (42.0, 47.8)	47.0 (43.7, 50.3)	
3-month	34.9 (32.0, 37.8)	33.5 (30.4, 36.6)	1.04 (0.86, 1.27)
6-month	39.3 (36.4, 42.2)	35.2 (32.1, 38.3)	1.18 (0.97, 1.42)

Abbreviations: CI, confidence interval; OR, odds ratio.

[a]: OR represent adjusted odds ratio for the intervention effect from the generalized estimating equation after adjusting for socio-demographic variables (caregiver's age, sex, education level, household income, frequency of using parenting apps, and recent learning about child injury prevention; child age and sex), outcome variables at baseline, and engagement with the interventions in the assigned group (number of logins, length of time using the app at each login, number of knowledge segments studied, number of knowledge segments bookmarked, and number of posted comments).

*: $P<0.05$.

Table MA3-4. Sensitivity analysis of primary and secondary outcomes based on per-protocol analysis

Outcome measure	Total		Intervention group		Control group	
	Rate	SE	Rate	SE	Rate	SE
**Unintentional injury incidence (%)**						
Baseline	9.5	0.7	9.6	1.0	9.4	1.0
3-month	7.6	0.6	8.1	0.9	7.1	0.9
6-month	7.9	0.6	8.2	0.9	7.5	0.9
**Score of attitudes toward unintentional injury prevention (mean)**						
Baseline	3.5	0.0	3.4	0.0	3.5	0.0
3-month	3.4	0.0	3.4	0.0	3.4	0.0
6-month	3.3	0.0	3.3	0.0	3.4	0.1
**Score of behavior toward unintentional injury prevention (mean)**						
Baseline	47.1	0.1	46.9	0.2	47.2	0.2
3-month	48.6	0.1	48.9	0.2	48.4	0.2
6-month	48.5	0.1	48.9	0.2	48.1	0.2

Note: Pre-protocol analysis was used to analyze data of participants who followed the protocol in the study time period, which was defined as individuals whose frequency of login to the app was ≤2 times or whose length of time using the app was ≤30 seconds.

Table MA3-5. Interaction between imbalanced baseline characteristics and primary outcome

Variables	OR (95% CI)
**Caregivers' sex × group (ref=male × group)**	
Female × group	0.81 (0.46, 1.43)
**Caregivers' age group × group (ref=≤29 years × group)**	
30-39 years × group	1.46 (0.76, 2.79)
40-49 years × group	2.96 (0.57, 15.38)
⩾50 years × group	0.80 (0.06, 10.25)
**Household income × group (ref= <1500 Yuan × group)**	
1500-3499 Yuan × group	8.36 (0.67, 104.58)
3500-5499 Yuan × group	2.71 (0.24, 30.96)
3500-5499 Yuan × group	6.35 (0.52, 77.32)
**Frequency of using parenting apps × group (ref= More than once a day × group)**	
Every two or three days × group	8.36 (0.67, 104.58)
Once a week × group	2.71 (0.24, 30.96)
Every two weeks or less × group	6.35 (0.52, 77.32)

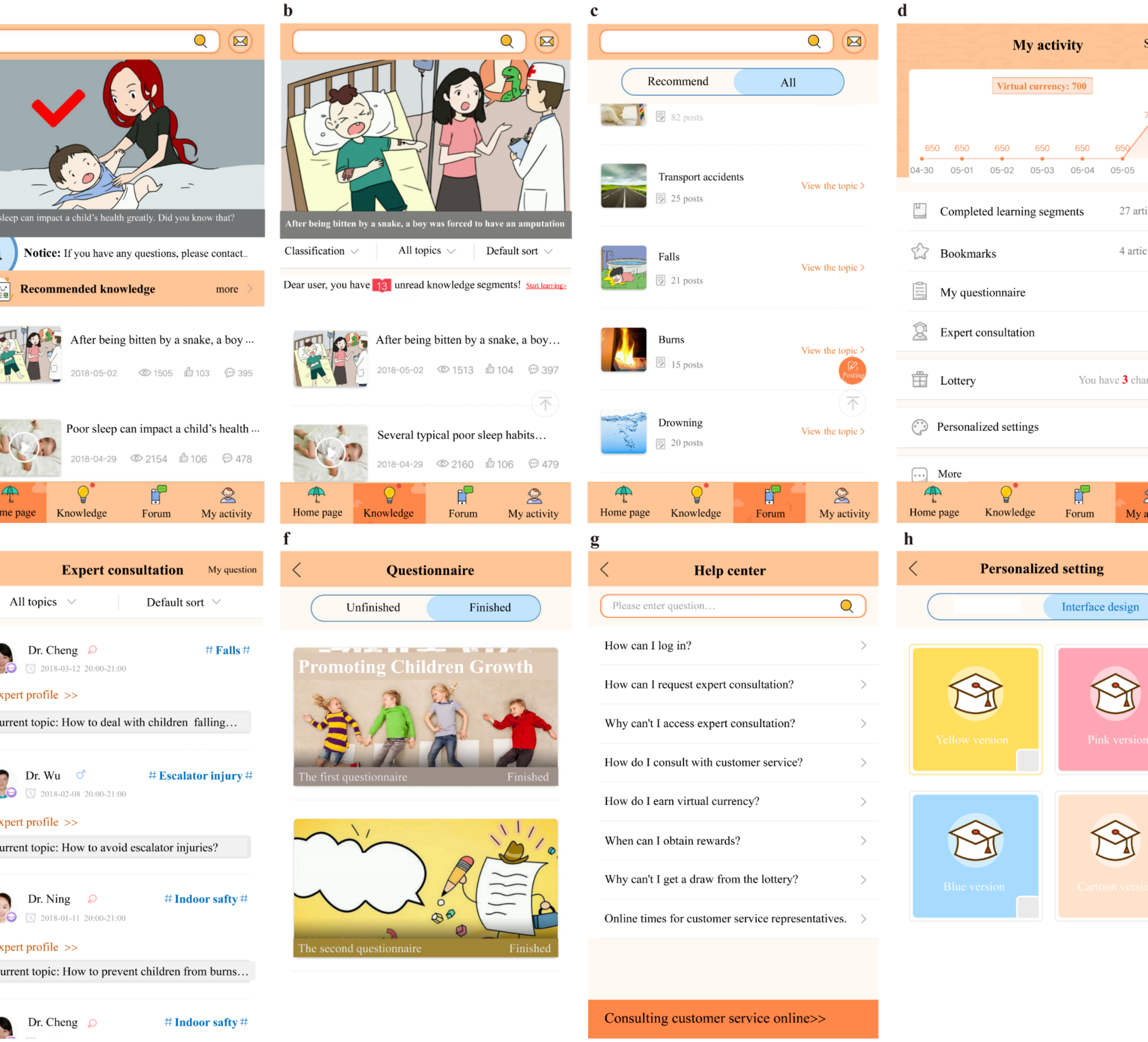

ure MA3-1. Translated homepage of the app intervention

Eight images within the figure were derived from the app "Bao Hu San." The app was been developed by the research team for unintentional injury prevention a hool students and was tested in this trial.

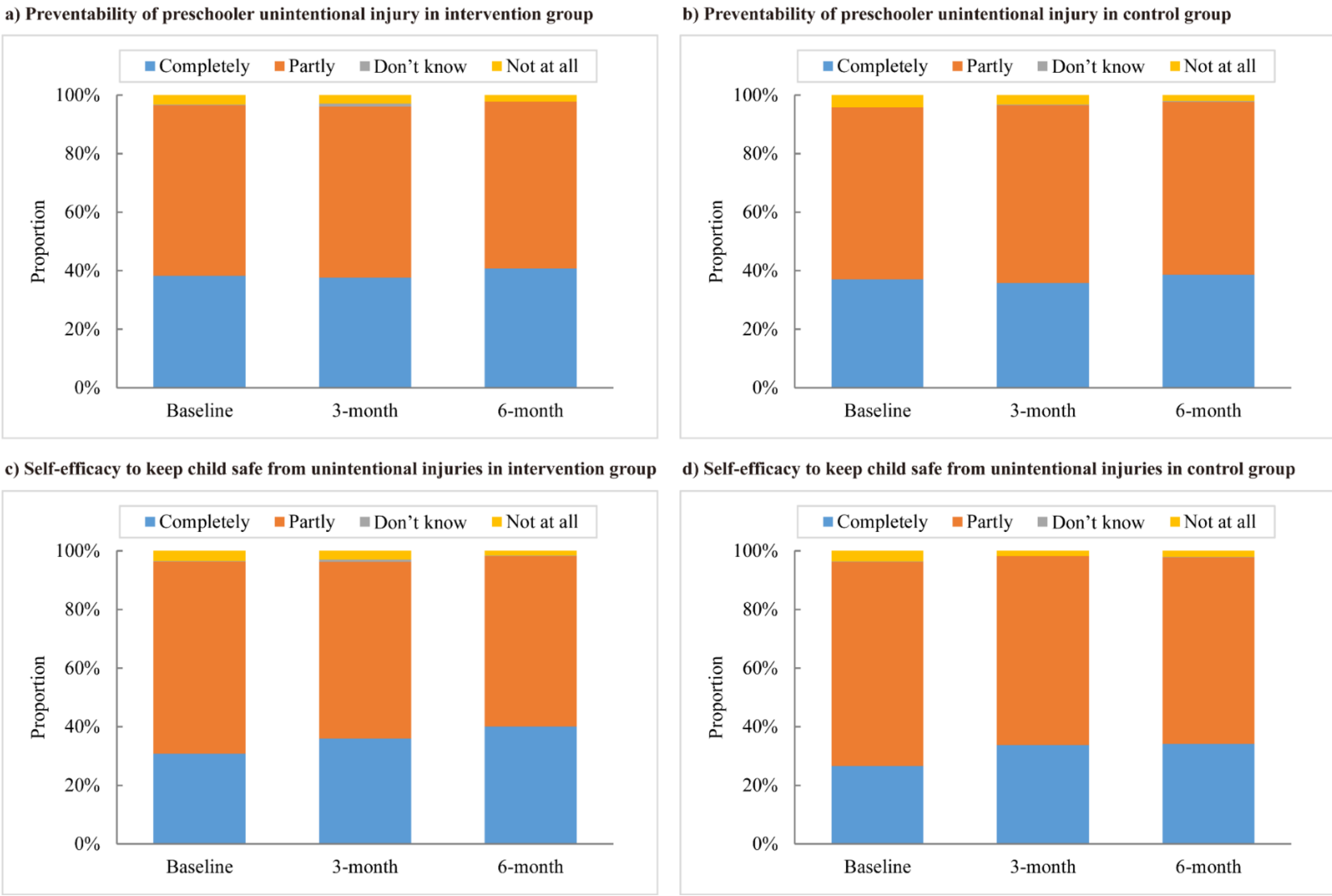

Figure MA3-2. Proportion of specific attitudes toward child unintentional injury prevention in the past week
